# Identification of gene signature for treatment response to guide precision oncology in clear-cell renal cell carcinoma

**DOI:** 10.1038/s41598-020-58804-y

**Published:** 2020-02-06

**Authors:** Ninadh M. D’Costa, Davide Cina, Raunak Shrestha, Robert H. Bell, Yen-Yi Lin, Hossein Asghari, Cesar U. Monjaras-Avila, Christian Kollmannsberger, Faraz Hach, Claudia I. Chavez-Munoz, Alan I. So

**Affiliations:** 1 Department of Urologic Sciences, Faculty of Medicine, University of British Columbia, Vancouver, BC Canada; 2 Vancouver Prostate Centre, Vancouver, BC Canada; 3 BC Cancer Agency, Vancouver, BC Canada; 4 School of Computing Science, Simon Fraser University, Burnaby, BC Canada

**Keywords:** Predictive markers, Renal cell carcinoma

## Abstract

Clear-cell renal cell carcinoma (ccRCC) is a common therapy resistant disease with aberrant angiogenic and immunosuppressive features. Patients with metastatic disease are treated with targeted therapies based on clinical features: low-risk patients are usually treated with anti-angiogenic drugs and intermediate/high-risk patients with immune therapy. However, there are no biomarkers available to guide treatment choice for these patients. A recently published phase II clinical trial observed a correlation between ccRCC patients’ clustering and their response to targeted therapy. However, the clustering of these groups was not distinct. Here, we analyzed the gene expression profile of 469 ccRCC patients, using featured selection technique, and have developed a refined 66-gene signature for improved sub-classification of patients. Moreover, we have identified a novel comprehensive expression profile to distinguish between migratory stromal and immune cells. Furthermore, the proposed 66-gene signature was validated using a different cohort of 64 ccRCC patients. These findings are foundational for the development of reliable biomarkers that may guide treatment decision-making and improve therapy response in ccRCC patients.

## Introduction

Clear-cell renal cell carcinoma (ccRCC) tumors have been reported to be highly angiogenic and with immunosuppressive features^1,2^. Recent publications show increased expression of the immune inhibitory ligand and receptors (PD-L1/CTLA4) on tumor cells and/or tumor-infiltrating immune cells^3,4^. Currently, tumor mutation burden is considered a predictive biomarker for response to immune checkpoint inhibitors (ICIs). However, research studies have shown that ccRCC has low mutational burden but highest immune infiltration score compared to other cancer types^5,6^. In a different study, a pan-cancer analysis found renal cell carcinomas (RCC) to have the highest proportion of indel mutations, which can increase tumor neoantigen abundance^7^. These anomalies in ccRCC make it the perfect platform to study dynamic biomarkers.

ccRCC patients with clinically localised tumor undergo partial or radical nephrectomy, but ~30% of the patients present with *de novo* metastatic disease^8^. Metastatic patients are usually treated with systemic therapies based on the clinical features^9^. The prognostic value of different risk stratification tools is limited to clinical and pathological features of the patients^10^. In Canada, International Metastatic Renal Cell Carcinoma Database Consortium (IMDC) risk model is applied with six clinical and laboratory factors: Karnofsky performance status, time of first-line targeted therapy from diagnosis, haemoglobin concentration, serum calcium concentration, neutrophil and platelet counts^9^. According to the IMDC risk stratification, low-risk metastatic RCC patients are usually treated with anti-angiogenic tyrosine kinase inhibitors (TKIs) and intermediate/high-risk patients with ICIs^11^. Risk stratification models based on gene expression pattern (both messenger and long non-coding RNA) in ccRCC have also proven to have strong prognostic values^12,13^. However, complete responses to therapy occur only in a small minority of patients^14^. Hence, there is an interest in the identification and development of treatment predictive biomarkers to enable precision oncology increasing drug response^5^.

Recently, a phase II clinical trial demonstrated a correlation between gene-expression based sub-classification of ccRCC patients, and their therapy response and prognosis^2^. Their study interrogated three biological axes, which, they hypothesized, play a role in the response to treatments. However, to our knowledge, these genes were specifically selected based on citations and not from empirical data analysis. In another phase III clinical trial, the authors analysed 1,500 genes and established that ccRCC tumor microenvironment play an important role in patients’ therapy response^15^. The latter study, however, did not explore a specific gene signature to sub-classify ccRCC patients.

Therefore, there is an unmet clinical need to identify a gene signature that will enable appropriate patient sub-classification and treatment decision-making for ccRCC patients. Here, we have analyzed the expression profiles of 469 ccRCC patients from The Cancer Genome Atlas (TCGA). All patients in this cohort have clear-cell histopathological classification (ccRCC) as they comprise approximately 70–75% of the RCC cases^8,16^. Data from primary tumors of both localised and metastatic cases are considered to develop our gene signature because it could potentially be the first step towards assessing gene expression pattern for all patients initially diagnosed with ccRCC. This can be a valuable tool to evaluate treatment response and prognosis of the patients regardless of their disease severity (metastatic or non-metastatic disease). Building on the foundation of personalised medicine, the current study identified a 66-gene signature that would accurately characterize ccRCC patients into appropriate sub-groups to guide precision oncology.

## Results

### Clinical characteristics of the ccRCC patients in our training and validation cohorts

To investigate the expression profile of ccRCC patients, we have used RNA sequencing data of 469 primary ccRCC samples from The Cancer Genome Atlas (TCGA) dataset in cBioPortal^17^. Dataset from Nature 2013 project was used as the training cohort (469 ccRCC patients) to determine the gene-signature for the study and a separate group of 64 ccRCC patients from the Provisional dataset, that did not match with the Nature 2013 dataset, was used as the validation cohort (schematic of workflow, Supplementary Fig. SF1). In the training cohort, there are 162 (34.5%) female and 307 (65.5%) male patients with median age of 63.7 years and 59.8 years, respectively. The validation cohort, on the other hand, has 26 (40.6%) female and 38 (59.4%) male patients with median age of 60.0 years and 62.5 years, respectively. As shown in Table 1, most samples were from localised tumors in both training (83.6%) and validation sets (46.2%) (Table 1).

**Table 1:** Patient demographics and clinical features of the ccRCC patients from The Cancer Genome Atlas (TCGA).

Training cohort, n = 469 patients			Validation cohort, n = 64 patients		
Characteristics	n	%	Characteristics	n	%
*Gender*			*Gender*		
Male	307	65.5	Male	38	59.4
Female	162	34.5	Female	26	40.6
*Median age (years)*			*Median age (years)*		
Male	59.8		Male	60.0	
Female	63.7		Female	62.5	
*Staging (TNM)*			*Staging (TNM)*		
I	224	47.8	I	43	67.2
II	48	10.2	II	9	14.1
III	116	24.7	III	7	10.9
IV	79	16.8	IV	4	6.3
Not reported	2	0.4	Not reported	1	1.6
*Tumor grade*			*Tumor grade*		
I	7	1.5	I	7	10.9
II	199	42.4	II	30	46.9
III	184	39.2	III	22	34.4
IV	73	15.6	IV	3	4.7
X	5	1.1	Not reported	2	3.1
Not reported	1	0.2	*Metastasis*		
			M0	30	46.9
*Metastasis*			M1	2	3.1
M0	392	83.6	MX	30	46.9
M1	77	16.4	Not reported	2	3.1

### Discovery of gene signature

**Figure 1:**
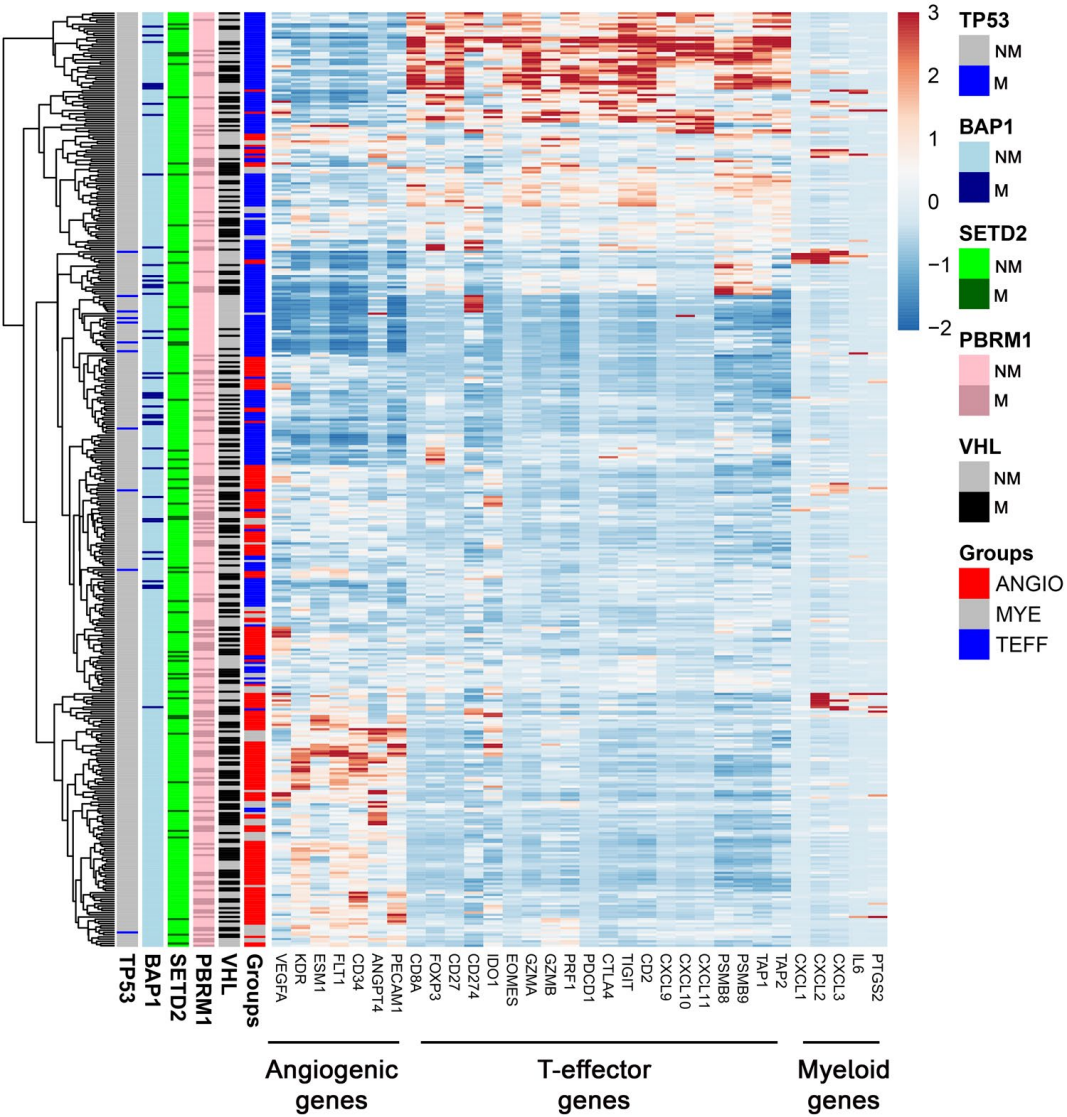
Heatmap confirming baseline ccRCC gene expression profile. Gene signature from IMmotion150 was used in the TCGA dataset of 469 ccRCC patients to generate a heatmap by unsupervised clustering based on Pearson correlation. Sub-classification of patients was based on k-nearest neighbour (kNN) classification method (k = 3). The heatmap shows that the ccRCC patients classified into 3 groups. Angio = 191 patients, T-eff = 222 patients and Myeloid = 56 patients. M = gene mutation, NM = no gene mutation.

Using the training dataset, we leveraged the IMmotion150 gene signature to perform unsupervised clustering on the gene-expression data using the Pearson correlation. We sub-classified the patients into three groups using k-nearest neighbour (kNN, k = 3) method and annotated samples as angiogenic, T-effector and myeloid inflammation (Angio = 191, T-eff = 222 and Myeloid = 56 patients). Our analysis corroborated the findings of the IMmotion150 study (Fig. 1).

To further identify the genes that distinguish the three clusters, we used featured selection technique on the global gene expression profile of the sub-classified patients. The genes were ranked based on their ability to best separate the patients into the labelled groups (“Angio”, “T-eff” and “Myeloid”). Next, we selected top 500 ranked genes for further investigation on the underlying biology (Supplementary Table ST1). In our top 500 genes, 21 genes matched and 8 genes were unique to IMmotion150 32-gene signature that were not identified by featured selection technique (Supplementary Fig. SF2, Table ST2). To understand the association of most frequently mutated genes in ccRCC, we analysed the mutation of VHL, BAP1, SETD2, PBRM1 and TP53 with the different patient clusters. Statistically significant association was found in TP53, BAP1 and PBRM1 mutation with both Angio and T-eff groups, respectively (Supplementary Table ST3). Interestingly, higher proportion of mutation for both TP53 and BAP1 was observed in T-eff group compared to Angio group, and PBRM1 mutation with Angio compared to T-eff group of ccRCC patients (Supplementary Table ST3).

### Refinement of gene signature

Our next target was to investigate the biological functions and pathways associated with the top 500 genes. “Myeloid” classification was removed because: (i) these genes did not affect the overall clustering pattern of the patients, (ii) the known myeloid genes from IMmotion150 were not among the top 500 genes and (iii) this cluster was not distinct from Angio and T-eff patient cohorts. Therefore, all the subsequent analyses were carried out considering Angio and T-eff clusters only.

**Figure 2:**
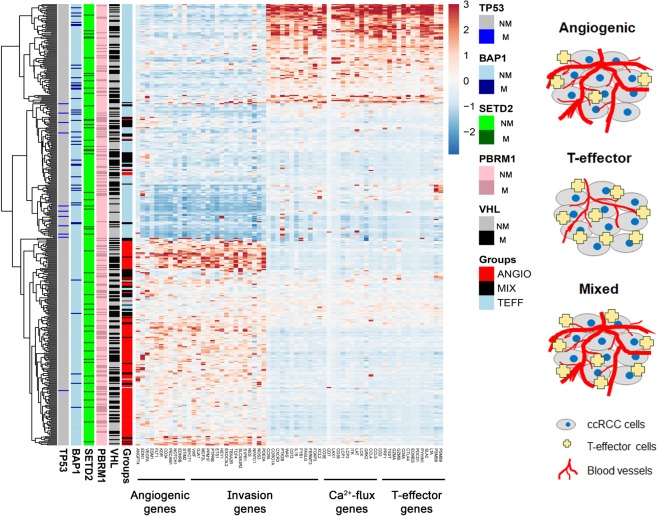
Heatmap with refined gene signature. The gene expression profile of the same 469 ccRCC patient cohort was used. Heatmap was generated using the new 66-gene signature by unsupervised clustering of patient with Pearson correlation. Sub-classification of patients was based on k-nearest neighbour (kNN) classification method (k = 3). Angio = 129, T-eff = 155 and Mixed = 131 patients. M = gene mutation, NM = no gene mutation.

The Ingenuity Pathway Analysis (IPA) of the 500 genes identified different molecular networks that demonstrated association with either Angio or T-eff genes. We prioritized genes involved in invasion and Ca^2+^-flux pathways because (i) these genes were in our top 500 genes ranked from the global expression profile of 20,483 genes using featured selection technique, (ii) IPA analysis determined these associated pathways based on the 500 gene list and (iii) their relevance in *de novo* metastatic ccRCC disease and IMDC clinical value, respectively (Supplementary Table ST2). The resulting 66-gene signature was then used to perform clustering of the patients based on their gene expression profile (Fig. 2). Interestingly, our results showed a distinct separation of the Invasion genes (gene = 30), where 18 genes clustered with Angio genes, the rest 12 genes clustered with T-eff genes. This is novel because a comprehensive expression profile to distinguish between migratory stromal and immune cells was not reported before.

Studies have previously reported the importance of Ca^2+^-flux related genes in immune cell activation^18^. Supporting this observation, our data showed tight clustering of Ca^2+^-flux genes with the T-effector genes. Furthermore, we identified a new “Mixed” cluster of patients who expressed genes from all the pathways (Angio, T-eff, Invasion, and Ca^2+^-flux). We also investigated the association of frequently mutated genes in ccRCC with our new patient clusters. Statistically significant association was found in TP53, BAP1 and PBRM1 mutation with both Angio and T-eff groups, respectively. TP53 and BAP1 mutations demonstrated an association with T-eff cluster, however, PBRM1 mutation associated to the Angio cluster (Supplementary Table ST4).

### Overall survival analysis of the patients

**Figure 3:**
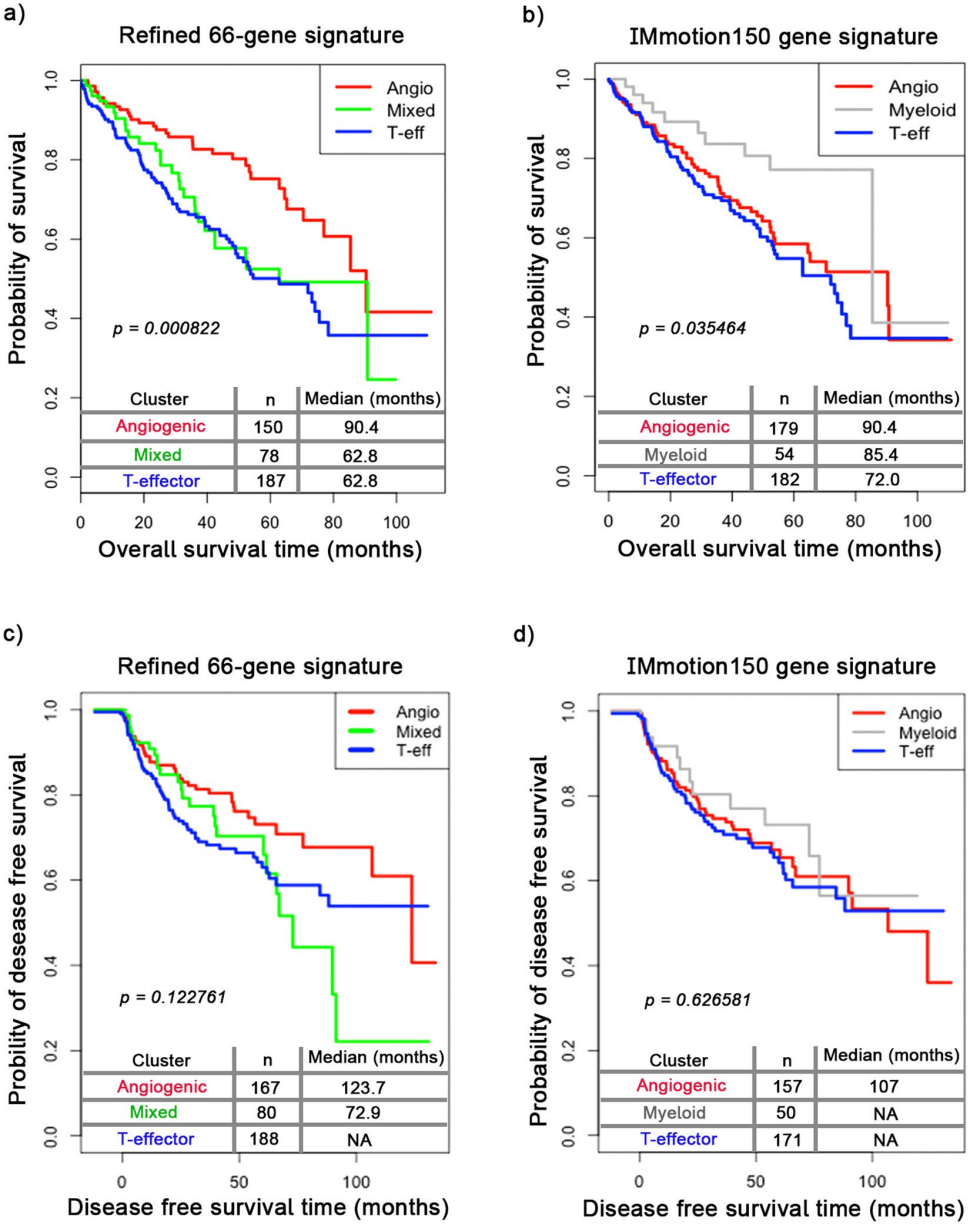
Kaplan-Meier analysis of overall and disease free survival time in patients. Overall survival time in our training dataset of 415 ccRCC patients was analyzed. (**a**) With our refined 66-gene signature, overall survival time in “Angio” patients (n = 150, median = 90.4 months) was found to be drastically higher than “T-eff” (n = 187, median = 62.8 months) and “Mixed” (n = 78, median = 62.8 months) group of patients (p = 0.00085016). (**b**) Overall survival time was not very different in “Angio” (n = 179, median = 90.4 months), “Myeloid” (n = 54, median = 85.4 months) and “T-eff” (n = 182, median = 72.0 months) patients based on IMmotion150 32-gene signature (p = 0.0196107). (**c**) Cox regression analysis of the disease free survival (DFS) in patients when 66-gene signature (p = 0.1227614) is used compared to (**d**) IMmotion150 gene signature (p = 0.6265809).

To investigate the association of the gene signature with the patients’ survival outcome, we analysed the overall survival time of 415 patients (survival data for 54 patients were not available). Using our gene signature, patients sub-classified as Angio cohort (n = 150, median = 90.4 months) had longer overall survival compared to T-eff (n = 187, median = 62.8 months) and Mixed (n = 78, median = 62.8 months) cohorts (Fig. 3a, p-value = 8.22 × 10^−4^). However, with IMmotion150 gene signature, a different survival outcome was observed in the same 415 patients. Here, the difference in overall survival of the Angio (n = 179, median = 90.4 months), T-eff (n = 182, median = 72 months) and Myeloid patients (n = 54, median = 85.4 months) was not as significant as with our gene signature (Fig. 3b, p-value = 3.54 × 10^−2^). This is probably due to re-classification of some patients when 66-gene signature is used compared to the IMmotion150 32-gene signature (Supplementary Table ST5). Our data suggests that the refined 66-gene signature is more robust and can accurately sub-classify ccRCC patients when correlating to its overall survival time data.

To assess the prognostic utility of our 66-gene signature with IMmotion150 gene signature, we have used Cox regression analysis on the disease free survival (DFS) time and status in the same cohorts of patients. Using our 66-gene signature, significantly high hazard ratio in the Mixed (exp (coef) = 1.7433, p = 0.0386) and T-effector (exp (coef) = 2.2091, p = 0.0201) groups was observed compared to the Angiogenic group. However, with IMmotion150 gene signature, no significant difference was observed in Myeloid (exp (coef) = 0.6513, p = 0.1985) and T-effector (exp (coef) = 0.6009, p = 0.1187) when compared to the Angiogenic group (Supplementary Table ST6, n = 378). A Kaplan-Meier analysis on the time to disease recurrence and progression shows a more distinct separation of cohorts with our 66-gene signature compared to IMmotion150 gene signature (Fig. 3c,d). Interestingly, when either invasion or Ca^2+^ -flux related genes are removed from the 66-gene signature, the strength of the clustering notably decreases (Supplementary Fig. SF3). This further provides evidence to the more accurate sub-classification ability of our proposed 66-gene signature.

### Establishing the reproducibility of 66-gene signature

**Figure 4:**
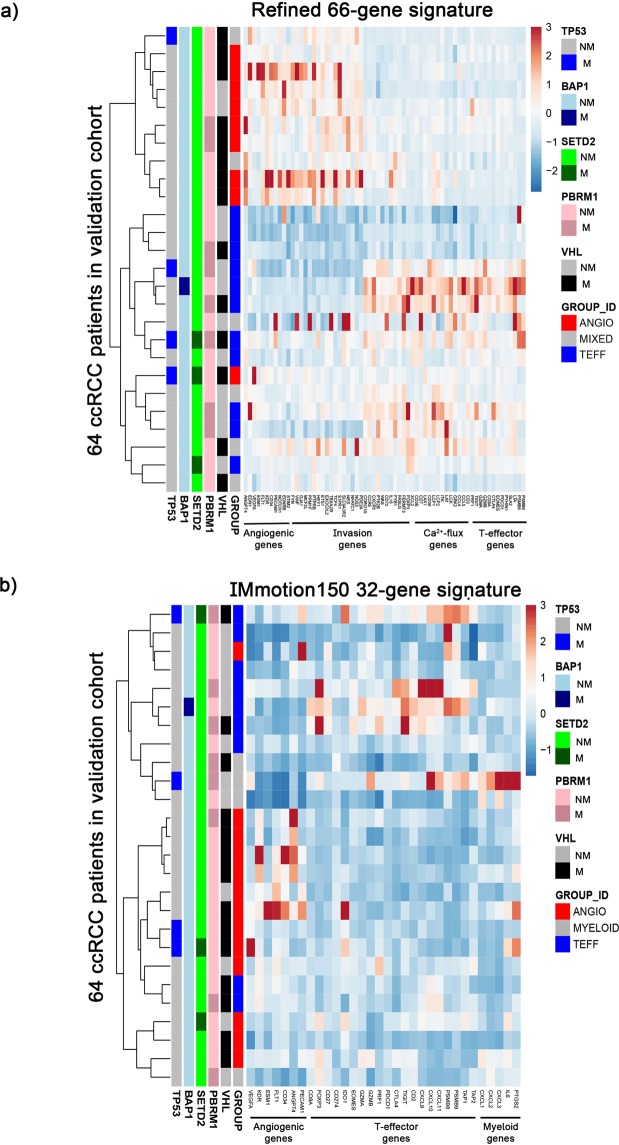
Confirmation of improved patient sub-classification with our 66-gene signature in a separate dataset. (**a**) Heatmap on the validation cohort of 64 ccRCC patients demonstrate a similar gene expression pattern like our previous training dataset and (**b**) expression pattern of the same cohort of patients when IMmotion150 gene signature is used. M = gene mutation, NM = no gene mutation, n = 64.

To further prove the robustness of our gene signature, we have validated the expression pattern observed with our 66-gene signature in a separate dataset of 64 ccRCC patients that were not included in the training dataset (n = 469, Nature 2013 dataset). Our data show that patients in the validation cohort also sub-classify into 3 separate clusters (Angio = 25, T-eff = 24 and Mixed = 15 patients) and demonstrate a similar gene expression pattern as the training dataset (Fig. 4a). The expression pattern with IMmotion150 gene signature, however, appeared very different from the training dataset on the same 64 ccRCC patients (Fig. 4b). The Kaplan-Meier analysis shows that our 66-gene signature has improved sub-classification of patients compared to that of IMmotion150 gene-signature. However, due to small number of patients in the validation set (n = 64), statistical significance was not achieved by either gene signatures (Supplementary Fig. SF4). This further establishes the reproducibility of our 66-gene signature in sub-classifying ccRCC patients.

## Discussions

As ccRCC is a highly vascular tumor, anti-angiogenic TKIs remained the long-standing first-line of treatment. However, previous studies demonstrated that ccRCC tumors are also rich with infiltrated immune cells and the patients with more immune cell infiltration have poor prognosis^2,3^. A recently published phase III clinical trial, COMPARZ, emphasized on the clinical significance of angiogenic and immune tumor microenvironment, and compared the treatment response of ccRCC patients to two TKIs (sunitinib and pazopanib)^15^. The authors categorized the patients into four clusters based on 1,500 genes and concluded that tumor microenvironment affects TKI response in ccRCC patients^15^. They also concluded that patients with enriched PBRM1 mutation associated with high angiogenic cluster, but BAP1 and TP53 mutations demonstrated a trend towards high immunogenic cluster. Our analysis on association of gene mutation and patient clustering corroborated their findings. However, ccRCC is a low mutation burden tumor and classification of patients based on gene mutation is not sufficient. Therefore, analysis of gene-expression profile is paramount for appropriate sub-classification of patients with ccRCC tumors.

Another recently published phase II clinical trial, IMmotion150, showed that patient outcomes were better when treated with targeted therapy based on patient genotypes^2^. The authors used the whole-transcriptome profile of 263 ccRCC patients and identified three clusters (angiogenic, T-effector cell and myeloid inflammation), and compared patient outcomes in response to sunitinib and atezolizumab/bevacizumab therapies^2^. With the same gene signature, we used the gene expression profile of 469 ccRCC patients and observed a similar clustering of patients. However, we did not observe a distinct patient clustering as presented in their publication^2^. Therefore, we used empirical data analysis to identify the genes that best separated the clusters. Consequently, we took advantage of feature selection techniques based on the variant of random forest on the expression profiles of 20,483 genes and discovered 500 ranked genes by their significance^19^. We removed myeloid sub-classification as the genes were not ranked in the top 500.

To understand the biological pathways involving the top 500 ranked genes, we used IPA analysis. The associated pathways identified by IPA analysis involved angiogenic, immunological, cell migration and invasion, and Ca^2+^-flux networks. Moreover, the invasion genes are known to be pivotal in metastatic disease and the Ca^2+^-flux genes have important clinical value by IMDC stratification^9,20^. We analysed the dataset of RCC patients with clear-cell histological subtype as it accounts for 70–75% of the RCC cases^8,16^. Our dataset was inclusive of primary ccRCC tumors of both localised and metastatic cases because the assessment of the primary tissue could be the starting point for evaluating the expression pattern of the patient (Angio, Teff or Mixed). This could, potentially, be a very critical step in determining the treatment options and the prognosis of the patient, whether or not they had metastatic disease.

In our gene signature, we observed that half of the identified invasion genes associated with the angiogenesis group and the other half with the T-effector group. This is novel because a distinct gene signature separating the migration of stromal cells for angiogenesis and of immune cells was not identified before. Immune cells are known to pave the way for tumor cell migration, invasion and metastasis, and their migration is mostly studied in the context of chemotaxis^21–23^. However, the expression profile in patients who might be more prone to immune cell mediated ccRCC disease progression has never been identified prior to this study. Furthermore, in COMPARZ, the authors were unable to assess tumor-infiltrating macrophage population due to the lack of a genetic signature^15^. Our finding here provides the gene signature that can be used in immune deconvolution.

Moreover, we analysed the association of gene signature with the overall survival time outcome in 469 ccRCC patients. We used both our 66-gene signature and IMmotion150 32-gene signature, and observed a remarkable change in Kaplan-Meier plots. With our 66-gene signature, the angiogenic stratified patients had significantly longer survival time compare to the other groups. This significant change could be due to the unavailability of ICIs as treatment options in 2013. Surprisingly, with IMmotion150 32-gene signature, there was not much difference in survival time among the three groups. This discrepancy could be explained by the difference in gene selection for patient sub-classification and the robustness of our gene signature. In a Cox regression analysis, our data shows a superior sub-classification ability of the refined 66-gene signature with a much lower likelihood ration (p = 0.1) compared to IMmotion150 (p = 0.7).

Furthermore, to establish the reproducibility of our 66-gene signature, we used the RNA-seq data of a separate cohort of 64 ccRCC patients from TCGA. Notably, we observed sub-classification of patients into 3 clusters with similar gene expression pattern in the patient groups. These results corroborated with our results from 469 ccRCC patients and established that our 66-gene signature can appropriately classify ccRCC patients to guide targeted therapy. However, future studies are necessary to investigate treatment response in ccRCC patients from each cluster when using our gene signature in order to validate precision treatment.

Altogether, our panel of 66-genes encompasses the crucial genes that have both therapeutic and clinical values in ccRCC. The proposed 66-gene signature, however, is not a risk stratification model to predict overall survival in patients. It is a clustering strategy of ccRCC patients based on their expression pattern to guide therapy-decision making and potentially improve patient outcome when treated with the appropriate systemic therapy.

Although there are strengths in our study, we also recognize some limitations. One limitation is that the treatment record and treatment outcome are not available in the TCGA data. Another caveat of the study is the small population size of the validation cohort. The other conundrum is the classification of the “Mixed” group and their potential treatment strategy that warrants further investigation.

In conclusion, we have refined the gene signature defined in IMmotion150 study by adding (i) new genes into the “Angio” and “T-eff” clusters for improved patient sub-classification and (ii) adding two newly identified pathways (Invasion and Ca^2+^-flux pathways). Our proposed strategy will enable the design of a panel of genes to better identify patients of appropriate genotype, and to improve treatment decision-making.

## Methods

### Patient data collection and clustering

Openly accessible data of the ccRCC patients was used for the study according to Human Subjects Protection and Data Access Policies by TCGA, National Cancer Institute (NCI) and the National Human Genome Research Institute (NHGRI). Data of de-identified ccRCC patients with informed consent was obtained according to TCGA Ethics & Policies, NCI and NHGRI. Patient demographics, RNA-sequencing (RNA-seq) and gene mutation data of ccRCC patients were obtained from the openly accessible TCGA-KIRC project in cBioPortal (Nature 2013 dataset, January 02, 2019, z-scored)^17^ following the TCGA Data Coordinating Centre (DCC), Open-Access Data Tier policy. To perform unsupervised classification, we used “pheatmap” function with Pearson correlation in R-studio for all the patient cohorts. Patients were clustered with the IMmotion150 32-gene signature using k-nearest neighbour (kNN) classification of 3 groups (k = 3): ccRCC patients with high expression pattern in T-effector genes were labeled as “T-eff”, high angiogenic genes as “Angio”, and high in myeloid inflammation genes as “Myeloid”. Using the proposed 66-gene signature, patients with high expression pattern in angiogenic genes were annotated as “Angio”, high with T-effector genes as “T-eff” and with mixed expression pattern as “Mixed”. For the validation cohort, Provisional dataset (534 ccRCC patients, March 18, 2019, cBioPortal) without the patients shared in Nature 2013 dataset was used (n = 64, one patient with unique sample ID was removed for computational convenience). mRNA expression data for the validation cohort was z-scored using “edgeR” in R-studio.

### Identification of novel gene signature

RNA-seq dataset of 469 ccRCC patients (Nature 2013, January 02, 2019) was used to apply feature selection for gene signature discovery. In this dataset, patients were first classified as “Angio”, “T-eff” and “Myeloid” groups proposed by IMmotion150 study using Pearson correlation, k-nearest neighbor method (k = 3). On these classified patients, we applied feature selection technique “Extremely Randomized Trees”, a variant of random forest, on the expression profiles of 20,483 genes^19,24^. This Extremely Randomized Trees classifier can be considered as a decision tree where each leaf node corresponds to a label (Angio, T-eff or Myeloid) and each internal node to a feature (mRNA z-score). The decision tree constructed algorithm used either information gain or gini index to measure the separateness in the clusters to each internal node and determine the best feature. The order of features selected in the decision tree was considered as their significance in the clustering step^25^. It was run in scikit-learn 0.21.2 and the number of trees in the forest was set to 1000. Features with “NA” values were converted to “0” (zero), as they would not affect the clustering results. The top 500 features (genes) that have the highest importance in the model are reported. Subsequently, Ingenuity Pathway Analysis (IPA, January 28, 2019) was used to identify the different molecular networks associated with these genes (QIAGEN Inc., https://www.qiagenbioinformatics.com/products/ingenuity-pathway-analysis).

### Statistical analysis

Clinical data on the consented ccRCC patients was obtained from TCGA-KIRK. All continuous variables were described with median and range values. Patients were sub-classified by unsupervised clustering on the gene-expression data using the Pearson correlation and k-nearest neighbour (kNN, k = 3) method. Overall survival time is defined as the date of nephrectomy to date of death. Recurrence/progression time is defined as the date from nephrectomy to the time of recurrence or progression of tumor burden. The association of the gene signature with patients’ survival outcome was measured using the median overall survival time and the time to recurrence/progression of the disease where appropriate. Median as “NA” was used when the median value was not obtained at 50 percentiles. The log-rank test in the “survival” R-package was used to generate the Kaplan-Meier plots. Death of a patient was used as the event in the survival analysis. Cox regression analysis was performed using “survival” R-package. The hazard ratio, likelihood ratio, and 95% confidence intervals (CIs) were obtained from recurrence/progression time outcome in patients. Gene mutation data was obtained from cBioPortal (Nature 2013 and Provisional). Patients clustered into groups were matched with the mutation dataset and their correlation was calculated based on χ^2^-test using “chisq.test” in R-studio. The p-values were corrected by Bonferroni correction method for multiple comparison using the R function “p.adjust”, and p-value < 0.05 was considered statistically significant.

## Supplementary information

Supplementary Data.
