# Supplementary material for: Identification of gene signature for treatment response to guide precision oncology in clear-cell renal cell carcinoma
Source: Sci Rep. 2020 Feb 6;10:2026. doi: 10.1038/s41598-020-58804-y (PMC7005147; doi:10.1038/s41598-020-58804-y)
Supplement: Supplementary file 1 — Supplementary Data. [file 41598_2020_58804_MOESM1_ESM.pdf]

Supplementary Data

Identification of gene signature for treatment response to guide precision oncology in clear-cell renal cell carcinoma.

Ninadh M. D'Costa[1,2], Davide Cina[1], Raunak Shrestha[1,2], Robert H Bell[1,2], Yen-Yi Lin[1,2], Hossein Asghari[2,4], Cesar Monjaras-Avila[2], Christian Kollmannsberger[3], Faraz Hach[1,2], Claudia Chavez-Munoz[1,2], *Alan I. So[1,2].

Affiliations:

[1]Department of Urologic Sciences, Faculty of Medicine, University of British Columbia, Vancouver, BC, Canada.

[2]Vancouver Prostate Centre, Vancouver, BC, Canada.

[3]BC Cancer Agency, Vancouver, BC, Canada

[4]School of Computing Science, Simon Fraser University, Burnaby, BC, Canada

***Corresponding author:**

Dr. Alan I. So, M.D.

Associate Professor and Senior Research Scientist

Department of Urologic Sciences, Faculty of Medicine, University of British Columbia

Vancouver Prostate Centre, 2660 Oak St., Vancouver, V6H 3Z6, BC, Canada

Email: dralanso@mail.ubc.ca, Phone: 604-875-4818, Fax: 604-875-5654

The authors declare no potential conflict of interests.

Content

1. **Supplementary Figure SF1:** Schematic diagram showing our study design.
2. **Supplementary Figure SF2:** Venn diagram showing the overlap in genes.
3. **Supplementary Figure SF3:** Kaplan-Meier plot of ccRCC patients with smaller gene panel.
4. **Supplementary Figure SF4:** Kaplan-Meier plot of the validation cohort of ccRCC patients.
5. **Supplementary Table ST1:** The top 500 ranked genes reported by featured selection technique.
6. **Supplementary Table ST2:** The genes selected for our 66-gene signature and their sources.
7. **Supplementary Table ST3:** Association of gene muation with Immotion150 patient clusters.
8. **Supplementary Table ST4:** Association of gene muation with 66-gene signature patient clusters.
9. **Supplementary Table ST5:** Patients classification pattern between the proposed 66-gene signature and IMmotion150 gene signature.
10. **Supplementary Table ST6:** Cox regression analysis of disease free survival time using the proposed 66-gene signature and IMmotion150 32-gene signature.

Supplementary Figure SF1

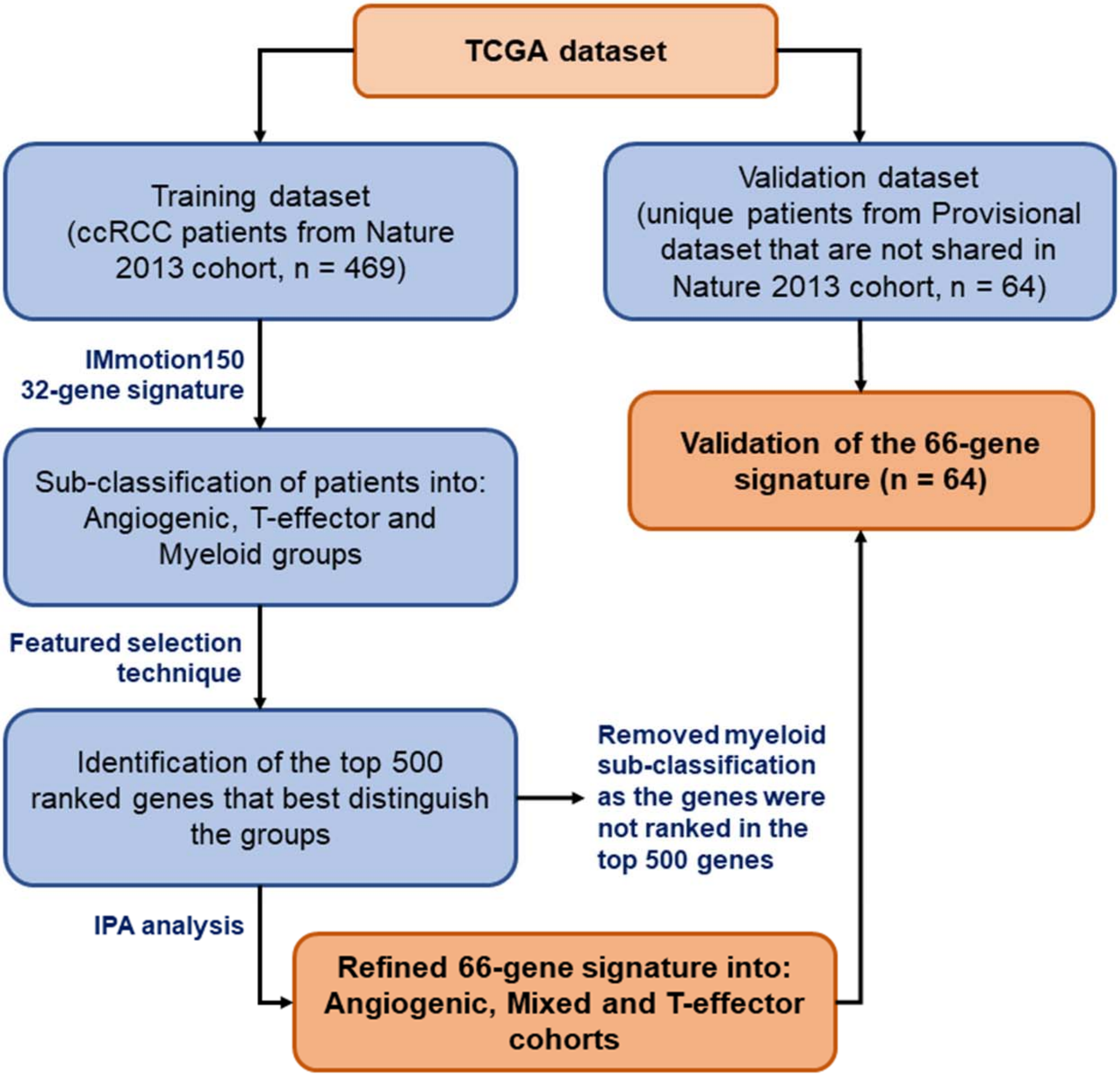

Supplementary Figure SF1: Schematic diagram showing our study design. A previously published 32-gene signature was used to sub-classify 469 ccRCC patients from TCGA. The global expression pattern of the sun-classified patients was analyzed to rank top 500 genes that distinguishes the clusters. IPA analysis on these 500 genes was used to select pathways involved in angiogenesis, cell migration and invasion, Ca^{2+}-flux and T-effectors cells. Consequently, a gene signature of 66 genes was defined that improved clustering of ccRCC patients. A separate cohort of 64 ccRCC patients was used to validate our refined 66-gene signature.

Supplementary Figure SF2

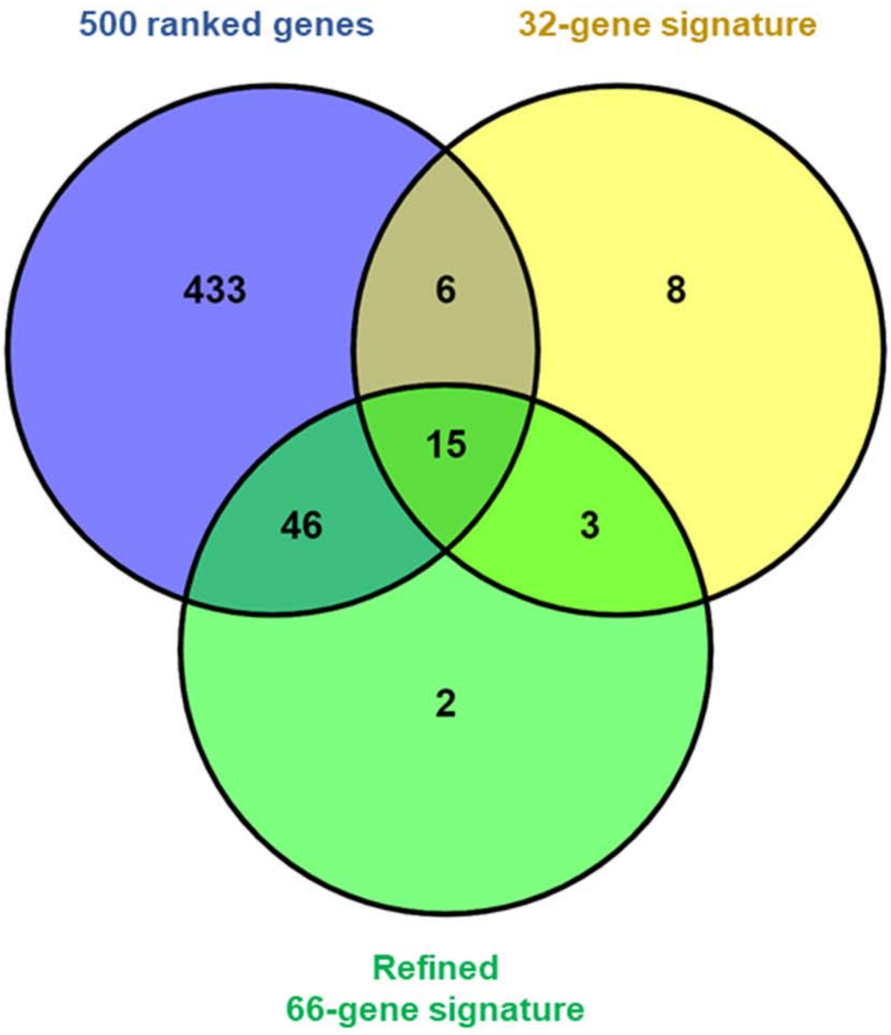

Supplementary Figure SF2: Venn diagram showing the overlap in genes. Featured selection technique based ranked 500 genes that best separated the patients into three different genotypes (angiogenic, T-effector and myeloid inflammation clusters) revealed 479 new genes (set blue only). Only 21 genes from IMmotion150 (intersection of set blue, yellow and green) matched with the 500 ranked genes. IPA analysis and relevant selection of Invasive and Ca2+-flux pathways of the 500 genes identified five associated new genes: 3 genes matched with IMmotion150 32-gene signature (intersection of set yellow and green only) and two entirely new genes (set green only). These analysis resulted in scrutinized selection of a 66-gene signature (set green).

Supplementary Figure SF3

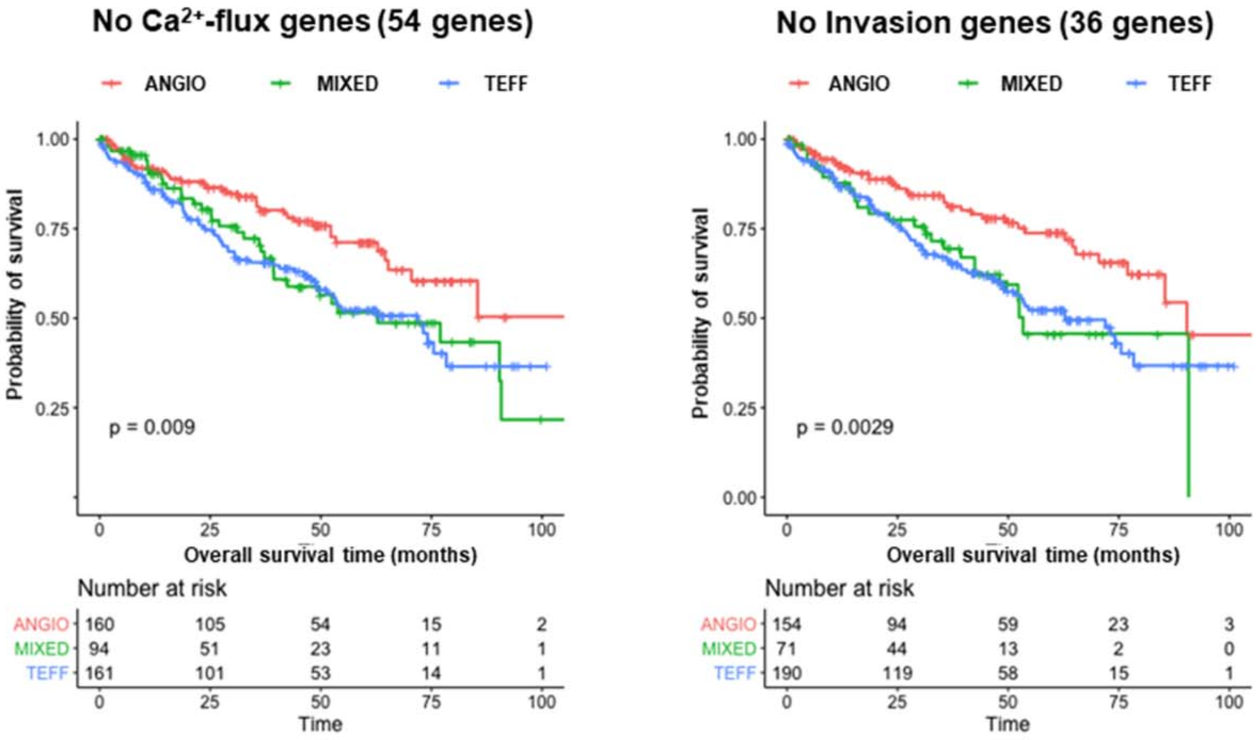

Supplementary Figure SF3: Kaplan-Meier plot of ccRCC patients with smaller gene panel. The proposed 66-gene signature is a comprehensive gene signature that significantly sub-classify ccRCC patients into angiogenic (ANGIO), T-effector (TEFF) and mixed (MIXED) clusters ($p = 0.0008$). Although statistically significant, when the invasion or Ca2+-flux related genes are removed, the strength of the classification decreases ($p = 0.009$ and $p = 0.0029$, respectively).

Supplementary Figure SF4

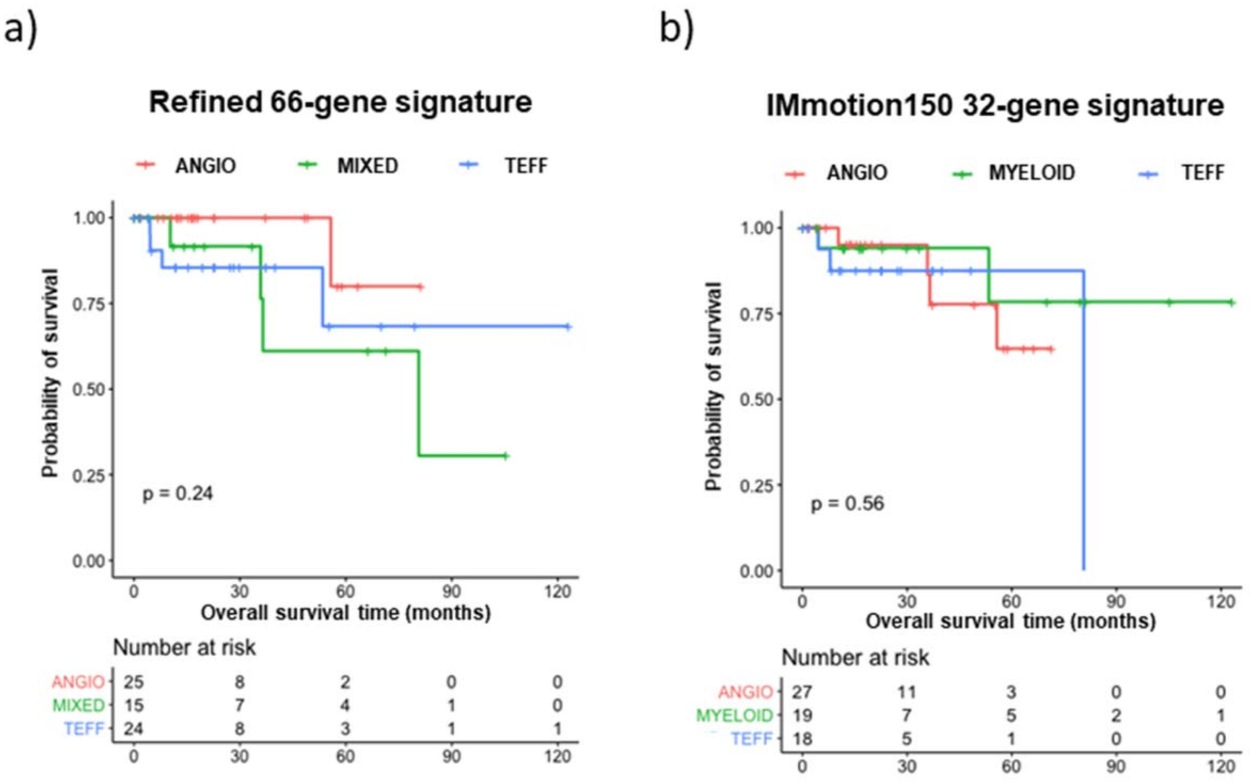

Supplementary Figure SF4: Kaplan-Meier plot of the validation cohort of ccRCC patients. A separate cohort of 64 ccRCC patients is used to validate the proposed 66-gene signature. a) With the refined 66-gene signature, a good separation of the groups is observed compared to b) IMmotion150 groups. Statistical significance is not achieved with either gene signature due to the small number of ccRCC patients in the validation cohort (n = 64).

Supplementary Table ST1: The top 500 ranked genes reported by featured selection

technique.

Entrez Gene ID	HUGO Gene Symbol	Global Rank	Featured Weight
149628	PYHIN1	1	0.0025
84174	SLA2	2	0.00235
914	CD2	3	0.00233
4049	LTA	4	0.00222
925	CD8A	5	0.00212
55423	SIRPG	6	0.00209
152789	JAKMIP1	7	0.00207
201633	TIGIT	8	0.00206
3932	LCK	9	0.00203
916	CD3E	10	0.00202
4753	NELL2	11	0.00202
939	CD27	12	0.00201
926	CD8B	13	0.002
128611	ZNF831	14	0.00184
27334	P2RY10	15	0.00182
5778	PTPN7	16	0.00181
3003	GZMK	17	0.00181
947	CD34	18	0.0018
9079	LDB2	19	0.00178
29851	ICOS	20	0.00171
4068	SH2D1A	21	0.0017
51294	PCDH12	22	0.00167
3643	INSR	23	0.00167
2078	ERG	24	0.00162
641700	ECSCR	25	0.00162
5698	PSMB9	26	0.00158
155038	GIMAP8	27	0.00157
6890	TAP1	28	0.00155
1234	CCR5	29	0.00155
2833	CXCR3	30	0.00154
3659	IRF1	31	0.00154
9760	TOX	32	0.00153
114836	SLAMF6	33	0.00153
9051	PSTPIP1	34	0.00153
2034	EPAS1	35	0.00151
3702	ITK	36	0.00151
3594	IL12RB1	37	0.00149
53347	UBASH3A	38	0.00148
115362	GBP5	39	0.00148
356	FASLG	40	0.00147
23177	CEP68	41	0.00143
1003	CDH5	42	0.00142

Entrez Gene ID	HUGO Gene Symbol	Global Rank	Featured Weight
3561	IL2RG	43	0.00141
9047	SH2D2A	44	0.0014
3604	TNFRSF9	45	0.00139
9744	ACAP1	46	0.00138
6397	SEC14L1	47	0.00137
56253	CRTAM	48	0.00136
374403	TBC1D10C	49	0.00135
8530	CST7	50	0.00134
919	CD247	51	0.00134
7075	TIE1	52	0.00133
64123	ELTD1	53	0.00131
11151	CORO1A	54	0.0013
80177	MYCT1	55	0.0013
6398	SECTM1	56	0.00129
22914	KLRK1	57	0.00128
79037	PVRIG	58	0.00128
9402	GRAP2	59	0.00128
917	CD3G	60	0.00127
2534	FYN	61	0.00126
90952	ESAM	62	0.00123
116071	BATF2	63	0.00123
3683	ITGAL	64	0.00123
1731	SEPT1	65	0.00122
154075	SAMD3	66	0.00122
3902	LAG3	67	0.00121
3112	HLA-DOB	68	0.00121
2113	ETS1	69	0.00121
7454	WAS	70	0.00121
5133	PDCD1	71	0.0012
4818	NKG7	72	0.00119
1E+08	LRRC70	73	0.00118
11184	MAP4K1	74	0.00115
2321	FLT1	75	0.00114
54873	PALMD	76	0.00113
3108	HLA-DMA	77	0.00113
4283	CXCL9	78	0.00112
401124	DTHD1	79	0.00111
2672	GFI1	80	0.00111
10225	CD96	81	0.00111
29116	MYLIP	82	0.00111
164118	TTC24	83	0.00111
3134	HLA-F	84	0.00108

Entrez Gene ID	HUGO Gene Symbol	Global Rank	Featured Weight
6352	CCL5	85	0.00108
84636	GPR174	86	0.00108
3001	GZMA	87	0.00108
2324	FLT4	88	0.00106
1012	CDH13	89	0.00106
50852	TRAT1	90	0.00106
972	CD74	91	0.00105
3111	HLA-DOA	92	0.00105
54440	SASH3	93	0.00104
25992	SNED1	94	0.00104
27240	SIT1	95	0.00104
6925	TCF4	96	0.00104
952	CD38	97	0.00103
6489	ST8SIA1	98	0.00103
5790	PTPRCAP	99	0.00103
124460	SNX20	100	0.00103
7450	VWF	101	0.00103
8320	EOMES	102	0.00103
1844	DUSP2	103	0.00101
2828	GPR4	104	0.00101
2841	GPR18	105	0.00101
80310	PDGFD	106	0.00101
5138	PDE2A	107	0.00101
1493	CTLA4	108	0.00101
115361	GBP4	109	0.001
5788	PTPRC	110	0.001
54538	ROBO4	111	0.00099
2633	GBP1	112	0.00099
5287	PIK3C2B	113	0.00098
6891	TAP2	114	0.00098
10252	SPRY1	115	0.00097
9495	AKAP5	116	0.00097
5175	PECAM1	117	0.00096
116835	HSPA12B	118	0.00096
79812	MMRN2	119	0.00094
441168	FAM26F	120	0.00094
7453	WARS	121	0.00094
22899	ARHGEF15	122	0.00093
6693	SPN	123	0.00093
7078	TIMP3	124	0.00092
6196	RPS6KA2	125	0.00092
4855	NOTCH4	126	0.00092
94	ACVRL1	127	0.00091
116159	CYYR1	128	0.00091
3458	IFNG	129	0.0009
170575	GIMAP1	130	0.00089
64411	ARAP3	131	0.00089
161198	CLEC14A	132	0.00089
3560	IL2RB	133	0.00089
83593	RASSF5	134	0.00088
51267	CLEC1A	135	0.00088
132332	TMEM155	136	0.00087
1901	S1PR1	137	0.00087
182	JAG1	138	0.00087
6373	CXCL11	139	0.00086
51513	ETV7	140	0.00086
83483	PLVAP	141	0.00085
63940	GPSM3	142	0.00085
399	RHOH	143	0.00084
6375	XCL1	144	0.00084
57572	DOCK6	145	0.00083
3791	KDR	146	0.00083
57823	SLAMF7	147	0.00083
54922	RASIP1	148	0.00082
22903	BTBD3	149	0.00082
10320	IKZF1	150	0.00082
9404	LPXN	151	0.00082
4858	NOVA2	152	0.00081
25780	RASGRP3	153	0.00081
375033	PEAR1	154	0.00081
3339	HSPG2	155	0.00081
51225	ABI3	156	0.00081
5551	PRF1	157	0.00081
10044	SH2D3C	158	0.00081
962	CD48	159	0.00079
64333	ARHGAP9	160	0.00079
653361	NCF1	161	0.00079
915	CD3D	162	0.00079
55303	GIMAP4	163	0.00079
79413	ZBED2	164	0.00079
639	PRDM1	165	0.00078
1521	CTSW	166	0.00078
83878	USHBP1	167	0.00078
85461	TANC1	168	0.00077
64919	BCL11B	169	0.00077
64218	SEMA4A	170	0.00077

Entrez Gene ID	HUGO Gene Symbol	Global Rank	Featured Weight
3587	IL10RA	171	0.00077
4005	LMO2	172	0.00077
22918	CD93	173	0.00077
54682	MANSC1	174	0.00076
2013	EMP2	175	0.00076
64321	SOX17	176	0.00076
79652	TMEM204	177	0.00075
5699	PSMB10	178	0.00075
2022	ENG	179	0.00074
3430	IFI35	180	0.00074
84230	LRRC8C	181	0.00074
83700	JAM3	182	0.00073
3714	JAG2	183	0.00073
752	FMNL1	184	0.00073
50619	DEF6	185	0.00073
200734	SPRED2	186	0.00073
60489	APOBEC3G	187	0.00073
126326	GIPC3	188	0.00073
9710	KIAA0355	189	0.00073
55340	GIMAP5	190	0.00073
7402	UTRN	191	0.00073
474344	GIMAP6	192	0.00072
5583	PRKCH	193	0.00072
8477	GPR65	194	0.00072
23348	DOCK9	195	0.00072
23129	PLXND1	196	0.00072
8404	SPARCL1	197	0.00072
51676	ASB2	198	0.00071
10385	BTN2A2	199	0.00071
257106	ARHGAP30	200	0.00071
10384	BTN3A3	201	0.00071
5696	PSMB8	202	0.00071
4430	MYO1B	203	0.00071
3936	LCP1	204	0.00071
64127	NOD2	205	0.00071
83439	TCF7L1	206	0.0007
2869	GRK5	207	0.0007
79961	DENND2D	208	0.0007
3113	HLA-DPA1	209	0.0007
131450	CD200R1	210	0.0007
54861	SNRK	211	0.00069
3106	HLA-B	212	0.00068
5155	PDGFB	213	0.00068

Entrez Gene ID	HUGO Gene Symbol	Global Rank	Featured Weight
2899	GRIK3	214	0.00068
6497	SKI	215	0.00068
54567	DLL4	216	0.00067
7010	TEK	217	0.00067
10395	DLC1	218	0.00067
10866	HCP5	219	0.00067
134265	AFAP1L1	220	0.00066
167465	ZNF366	221	0.00066
10663	CXCR6	222	0.00066
8578	SCARF1	223	0.00066
51744	CD244	224	0.00066
3627	CXCL10	225	0.00065
255877	BCL6B	226	0.00065
3707	ITPKB	227	0.00065
3133	HLA-E	228	0.00065
11119	BTN3A1	229	0.00065
126375	ZNF792	230	0.00064
3384	ICAM2	231	0.00064
146206	RLTPR	232	0.00064
57477	SHROOM4	233	0.00064
6846	XCL2	234	0.00064
130132	RFTN2	235	0.00064
3122	HLA-DRA	236	0.00063
6935	ZEB1	237	0.00063
10870	HCST	238	0.00063
2533	FYB	239	0.00063
57568	SIPA1L2	240	0.00063
27115	PDE7B	241	0.00062
55534	MAML3	242	0.00062
169611	OLFML2A	243	0.00062
164668	APOBEC3H	244	0.00062
9046	DOK2	245	0.00062
3603	IL16	246	0.00062
2643	GCH1	247	0.00061
1948	EFNB2	248	0.00061
83857	TMTC1	249	0.00061
152273	FGD5	250	0.00061
83872	HMCN1	251	0.0006
22806	IKZF3	252	0.0006
6753	SSTR3	253	0.0006
79905	TMC7	254	0.0006
9771	RAPGEF5	255	0.0006
23046	KIF21B	256	0.0006

Entrez Gene ID	HUGO Gene Symbol	Global Rank	Featured Weight
2697	GJA1	257	0.00059
51291	GMIP	258	0.00059
2313	FLI1	259	0.00059
4947	OAZ2	260	0.00059
5362	PLXNA2	261	0.00059
286530	P2RY8	262	0.00059
26191	PTPN22	263	0.00059
1910	EDNRB	264	0.00059
29941	PKN3	265	0.00058
256435	ST6GALNAC3	266	0.00058
80774	LIMD2	267	0.00058
151888	BTLA	268	0.00058
30009	TBX21	269	0.00058
5420	PODXL	270	0.00058
6772	STAT1	271	0.00058
83706	FERMT3	272	0.00057
2004	ELK3	273	0.00057
135	ADORA2A	274	0.00056
9223	MAGI1	275	0.00056
80833	APOL3	276	0.00056
10008	KCNE3	277	0.00056
1846	DUSP4	278	0.00056
147138	TMC8	279	0.00055
2934	GSN	280	0.00055
5367	PMCH	281	0.00055
4046	LSP1	282	0.00055
497189	TIFAB	283	0.00054
23075	SWAP70	284	0.00054
25927	CNRIP1	285	0.00054
2634	GBP2	286	0.00054
10068	IL18BP	287	0.00053
51816	CECR1	288	0.00053
23225	NUP210	289	0.00053
80709	AKNA	290	0.00053
6405	SEMA3F	291	0.00053
3937	LCP2	292	0.00053
5787	PTPRB	293	0.00053
225	ABCD2	294	0.00053
5777	PTPN6	295	0.00053
9537	TP53I11	296	0.00052
149699	GTSF1L	297	0.00052
4208	MEF2C	298	0.00052
8642	DCHS1	299	0.00052

Entrez Gene ID	HUGO Gene Symbol	Global Rank	Featured Weight
3672	ITGA1	300	0.00052
156	ADRBK1	301	0.00052
5920	RARRES3	302	0.00051
55013	CCDC109B	303	0.00051
257068	PLCXD2	304	0.00051
8503	PIK3R3	305	0.00051
27253	PCDH17	306	0.00051
8692	HYAL2	307	0.00051
10538	BATF	308	0.00051
114769	CARD16	309	0.00051
92241	RCSD1	310	0.0005
3563	IL3RA	311	0.0005
57088	PLSCR4	312	0.0005
7126	TNFAIP1	313	0.00049
387357	THEMIS	314	0.00049
2701	GJA4	315	0.00049
80308	FLAD1	316	0.00049
256949	KANK3	317	0.00049
4261	CIITA	318	0.00049
2563	GABRD	319	0.00049
10537	UBD	320	0.00048
6351	CCL4	321	0.00048
10052	GJC1	322	0.00048
4851	NOTCH1	323	0.00048
23157	43714	324	0.00048
1849	DUSP7	325	0.00048
26207	PITPNC1	326	0.00048
57381	RHOJ	327	0.00048
3695	ITGB7	328	0.00048
160365	CLECL1	329	0.00048
23328	SASH1	330	0.00048
3105	HLA-A	331	0.00048
4162	MCAM	332	0.00048
5880	RAC2	333	0.00047
84225	ZMYND15	334	0.00047
221184	CPNE2	335	0.00047
3136	HLA-H	336	0.00047
2	A2M	337	0.00047
11170	FAM107A	338	0.00047
2887	GRB10	339	0.00047
8611	PPAP2A	340	0.00047
9647	PPM1F	341	0.00047
3107	HLA-C	342	0.00047

Entrez Gene ID	HUGO Gene Symbol	Global Rank	Featured Weight
114800	CCDC85A	343	0.00047
10906	TRAFD1	344	0.00047
56548	CHST7	345	0.00047
126669	SHE	346	0.00047
11082	ESM1	347	0.00046
90332	EXOC3L2	348	0.00046
196883	ADCY4	349	0.00046
7881	KCNAB1	350	0.00046
27145	FILIP1	351	0.00046
11118	BTN3A2	352	0.00046
23526	HMHA1	353	0.00046
140564	APOBEC3D	354	0.00046
84969	TOX2	355	0.00046
115352	FCRL3	356	0.00045
143903	LAYN	357	0.00045
5993	RFX5	358	0.00045
9749	PHACTR2	359	0.00045
9246	UBE2L6	360	0.00045
1879	EBF1	361	0.00045
117248	GALNTL2	362	0.00045
80342	TRAF3IP3	363	0.00045
3662	IRF4	364	0.00044
55901	THSD1	365	0.00044
10563	CXCL13	366	0.00044
140685	ZBTB46	367	0.00044
57153	SLC44A2	368	0.00044
4542	MYO1F	369	0.00044
64926	RASAL3	370	0.00044
54438	GFOD1	371	0.00044
90627	STARD13	372	0.00043
5476	CTSA	373	0.00043
9351	SLC9A3R2	374	0.00043
114614	MIR155HG	375	0.00043
2050	EPHB4	376	0.00043
26157	GIMAP2	377	0.00043
8490	RGS5	378	0.00043
6737	TRIM21	379	0.00043
6688	SPI1	380	0.00043
654817	NCF1C	381	0.00043
51379	CRLF3	382	0.00043
9891	NUAK1	383	0.00043
10203	CALCRL	384	0.00042
1906	EDN1	385	0.00042
9411	ARHGAP29	386	0.00042
2207	FCER1G	387	0.00042
22807	IKZF2	388	0.00042
6297	SALL2	389	0.00042
10763	NES	390	0.00042
8829	NRP1	391	0.00042
9580	SOX13	392	0.00042
441478	NRARP	393	0.00041
3092	HIP1	394	0.00041
7082	TJP1	395	0.00041
51021	MRPS16	396	0.00041
3116	HLA-DPB2	397	0.00041
4811	NID1	398	0.00041
256364	EML3	399	0.00041
3109	HLA-DMB	400	0.00041
387923	SERP2	401	0.00041
153579	BTNL9	402	0.00041
51056	LAP3	403	0.00041
963	CD53	404	0.00041
197358	NLRC3	405	0.00041
2124	EVI2B	406	0.00041
23263	MCF2L	407	0.0004
9828	ARHGEF17	408	0.0004
54847	SIDT1	409	0.0004
10268	RAMP3	410	0.0004
81848	SPRY4	411	0.0004
7185	TRAF1	412	0.0004
25960	GPR124	413	0.0004
924	CD7	414	0.00039
3720	JARID2	415	0.00039
342926	ZNF677	416	0.00039
8291	DYSF	417	0.00039
482	ATP1B2	418	0.00039
4846	NOS3	419	0.00039
3340	NDST1	420	0.00039
27040	LAT	421	0.00039
8111	GPR68	422	0.00039
5293	PIK3CD	423	0.00038
1464	CSPG4	424	0.00038
26262	TSPAN17	425	0.00038
5939	RBMS2	426	0.00038
864	RUNX3	427	0.00038
64222	TOR3A	428	0.00038

Entrez Gene ID	HUGO Gene Symbol	Global Rank	Featured Weight
11015	KDELR3	429	0.00038
3139	HLA-L	430	0.00038
81619	TSPAN14	431	0.00038
430	ASCL2	432	0.00038
5579	PRKCB	433	0.00037
4772	NFATC1	434	0.00037
221395	GPR116	435	0.00037
222553	SLC35F1	436	0.00037
254263	CNIH2	437	0.00037
83595	SOX7	438	0.00037
606724	LOC606724	439	0.00037
23616	SH3BP1	440	0.00037
51177	PLEKHO1	441	0.00037
81575	APOLD1	442	0.00037
51313	FAM198B	443	0.00036
79626	TNFAIP8L2	444	0.00036
57480	PLEKHG1	445	0.00036
6892	TAPBP	446	0.00036
201294	UNC13D	447	0.00036
6525	SMTN	448	0.00036
28951	TRIB2	449	0.00036
54331	GNG2	450	0.00036
2624	GATA2	451	0.00036
51296	SLC15A3	452	0.00036
445815	PALM2-AKAP2	453	0.00035
441027	TMEM150C	454	0.00035
260293	CYP4X1	455	0.00035
10379	IRF9	456	0.00035
57620	STIM2	457	0.00035
79843	FAM124B	458	0.00035
168537	GIMAP7	459	0.00035
9467	SH3BP5	460	0.00035
57496	MKL2	461	0.00035
7535	ZAP70	462	0.00035
6915	TBXA2R	463	0.00035
3632	INPP5A	464	0.00034
5937	RBMS1	465	0.00034
3115	HLA-DPB1	466	0.00034
923	CD6	467	0.00034
50943	FOXP3	468	0.00034
23637	RABGAP1	469	0.00034
283420	CLEC9A	470	0.00034
7092	TLL1	471	0.00034
219285	SAMD9L	472	0.00034
10420	TESK2	473	0.00034
9997	SCO2	474	0.00034
11098	PRSS23	475	0.00034
7102	TSPAN7	476	0.00033
26524	LATS2	477	0.00033
7456	WIPF1	478	0.00033
6867	TACC1	479	0.00033
30844	EHD4	480	0.00033
5168	ENPP2	481	0.00033
23462	HEY1	482	0.00033
912	CD1D	483	0.00033
165186	FAM179A	484	0.00033
132160	PPM1M	485	0.00033
3135	HLA-G	486	0.00033
23214	XPO6	487	0.00033
10105	PPIF	488	0.00033
10194	TSHZ1	489	0.00033
971	CD72	490	0.00033
54900	LAX1	491	0.00033
2185	PTK2B	492	0.00033
6345	SRL	493	0.00032
158376	LOC158376	494	0.00032
9217	VAPB	495	0.00032
801	CALM1	496	0.00032
4804	NGFR	497	0.00032
10039	PARP3	498	0.00032
6793	STK10	499	0.00032
285	ANGPT2	500	0.00032

Supplementary Table ST2: The genes selected for our 66-gene signature and their sources.

66-gene signature	Rank	Source
ANGPT4	NA	IMmotion150
EDN1	385	
VEGFA	NA	IMmotion150
ESM1	347	
FLT1	75	
KDR	146	
CD34	18	
PECAM1	117	
NOTCH1	323	
EDNRB	264	
STIM2	457	
FYN	61	
VWF	101	
GJA1	257	
MCF2L	407	
PPM1F	314	
PTPRB	293	
HEY1	482	
ETS1	69	
EXOC3L2	348	
TBXA2R	463	
TCF4	96	
S1PR1	137	
SLC9A3R2	374	
NES	390	
NFATC1	434	
NOS3	419	
PDE2A	107	
CORO1A	54	
CCR5	29	
CXCR3	30	
PTK2B	492	
WAS	70	

66-gene signature	Rank	Source
CD72	490	
IL16	246	
FYB1	NA	IPA: Cell Movement network (Invasion)
FASLG	40	
FERMT3	272	
FOXP3	468	
XCL2	234	
CD3E	10	
CD7	414	
LAX1	491	
CD38	97	
LCP1	204	
LCP2	292	
ITK	36	
LAT	421	
LCK	9	
GRK2	NA	IPA: Ca2+ flux network
CCL4	321	
CCL5	85	
CD2	3	
PRF1	157	
TIGIT	8	
GZMA	87	
GZMB	NA	IMmotion150
CD8A	5	
CTLA4	108	
EOMES	102	
PDCD1	71	
PYHIN1	1	
SLA2	2	
LTA	4	
PSMB8	202	
PSMB9	26	

Supplementary Table ST3: Association of gene muation with Immotion150 patient clusters.

	Mutated gene	Cluster	p-value	Bonferroni corrected p-value	Mutated, Cluster	Not mutated, Cluster	Mutated, Non-Cluster	Not mutated, Non-Cluster
IMmotion150 32-gene signature	**VHL**	**ANGIO**	0.1592	1	93	79	117	134
	**VHL**	**TEFF**	0.0446	0.7136	87	110	123	103
	**VHL**	**MYE**	0.43285	1	30	24	180	189
	**TP53**	**ANGIO**	0.01347	0.21552	0	172	11	240
	**TP53**	**TEFF**	0.00734	0.11744	10	187	1	225
	**TP53**	**MYE**	1	1	1	53	10	359
	**BAP1**	**ANGIO**	0.00137	0.0218464	6	166	33	218
	**BAP1**	**TEFF**	3.2E-05	0.00051699	31	166	8	218
	**BAP1**	**MYE**	0.21191	1	2	52	37	332
	**SETD2**	**ANGIO**	1	1	20	152	28	223
	**SETD2**	**TEFF**	1	1	22	175	26	200
	**SETD2**	**MYE**	1	1	6	48	42	327
	**PBRM1**	**ANGIO**	0.00122	0.0194672	68	104	61	190
	**PBRM1**	**TEFF**	8.4E-05	0.00134075	41	156	88	138
	**PBRM1**	**MYE**	0.3373	1	20	34	109	260

Supplementary Table ST4: Association of gene mutation with 66-gene signature patient clusters.

	Mutated gene	Cluster	p-value	Bonferroni corrected p-value	Mutated, Cluster	Not mutated, Cluster	Mutated, Non-Cluster	Not mutated, Non-Cluster
Refined 66-gene signature	VHL	TEFF	0.00555	0.088752	97	127	114	85
	VHL	ANGIO	0.0341	0.54552	97	75	114	137
	VHL	MIX	0.22777	1	17	10	194	202
	TP53	TEFF	0.00422	0.06746624	11	213	0	199
	TP53	ANGIO	0.01348	0.2156112	0	172	11	240
	TP53	MIX	0.80056	1	0	27	11	385
	BAP1	TEFF	0.00823	0.13172304	29	216	10	189
	BAP1	ANGIO	0.0001	0.00162955	4	168	35	216
	BAP1	MIX	0.03847	0.61544	6	21	33	363
	SETD2	TEFF	0.55572	1	23	201	25	174
	SETD2	ANGIO	0.75077	1	18	154	30	221
	SETD2	MIX	0.03117	0.4987616	7	20	41	355
	PBRM1	TEFF	0.00347	0.0555616	54	170	75	124
	PBRM1	ANGIO	0.00503	0.080524	66	106	63	188
	PBRM1	MIX	0.90852	1	9	18	120	276

Supplementary Table ST5: Patients classification pattern between the proposed 66-gene signature and IMmotion150 gene signature.

Matched classification of patients		
Groups	n	%
Angiogenic	124	26.4
T-effector	195	41.6
Total	319	68.0

Re-classified patients			
66-gene Groups	IMmotion150 Groups	n	%
Angiogenic	T-effector	9	1.9
T-effector	Angiogenic	18	3.8
Mixed	Angiogenic	50	10.7
Mixed	T-effector	18	3.8
Mixed	Myeloid	14	3.0
Angiogenic	Myeloid	29	6.2
T-effector	Myeloid	12	2.6
Total		150	32.0

Supplementary Table ST6: Cox regression analysis of disease free survival time using the proposed 66-gene signature and IMmotion150 32-gene signature.

**Refined 66-gene signature, n= 378**	coef	exp (coef)	se (coef)	z	p-value
Compared to Angio					
Mixed	0.556	1.743	0.269	2.068	*0.039
T-eff	0.793	2.209	0.341	2.325	*0.020
Likelihood ratio test					0.100
Wald test					0.100
Score (logrank)					0.100

**IMmotion150 32-gene signature, n=378**	coef	exp (coef)	se (coef)	z	p-value
Compared to Angio					
Myeloid	-0.429	0.651	0.334	-1.286	0.199
T-eff	-0.509	0.601	0.326	-1.560	0.119
Likelihood ratio test					0.700
Wald test					0.700
Score (logrank)					0.700
